# Error margin analysis for feature gene extraction

**DOI:** 10.1186/1471-2105-11-241

**Published:** 2010-05-11

**Authors:** Chi Kin Chow, Hai Long Zhu, Jessica Lacy, Winston P Kuo

**Affiliations:** 1 Research institute of Innovative Products and Technologies, The Hong Kong Polytechnic University, Hong Kong SAR, PR China; 2 Laboratory for Innovative Translational Technologies, Harvard Medical School, Boston, MA, USA

## Abstract

**Background:**

Feature gene extraction is a fundamental issue in microarray-based biomarker discovery. It is normally treated as an optimization problem of finding the best predictive feature genes that can effectively and stably discriminate distinct types of disease conditions, e.g. tumors and normals. Since gene microarray data normally involves thousands of genes at, tens or hundreds of samples, the gene extraction process may fall into local optimums if the gene set is optimized according to the maximization of classification accuracy of the classifier built from it.

**Results:**

In this paper, we propose a novel gene extraction method of error margin analysis to optimize the feature genes. The proposed algorithm has been tested upon one synthetic dataset and two real microarray datasets. Meanwhile, it has been compared with five existing gene extraction algorithms on each dataset. On the synthetic dataset, the results show that the feature set extracted by our algorithm is the closest to the actual gene set. For the two real datasets, our algorithm is superior in terms of balancing the size and the validation accuracy of the resultant gene set when comparing to other algorithms.

**Conclusion:**

Because of its distinct features, error margin analysis method can stably extract the relevant feature genes from microarray data for high-performance classification.

## Background

Gene expression data commonly involve thousands of genes at, tens or hundreds of samples. In order to reduce the computation cost and complexity of the classification, feature extraction on gene expression pattern is necessary. The objective of feature gene extraction process is to select the gene set that can be used to effectively and stably discriminate distinct types of disease statuses, e.g. tumors and normals.

According to the terminology proposed in [1], one of the major approaches available in feature selection is *filter model*. It uses statistical techniques over the training patterns to "filter out" irrelevant features. Yet the "filtering" process can be further divided to forward selection and backward elimination. In forward selection [2], variables are progressively incorporated into larger and larger subsets, whereas in backward elimination, one starts with the set of all variables and progressively eliminates the least relevant ones. In the field of bioinformatics, there is a belief that the class of a gene expression pattern, either *normal *or *cancerous*, correlates to the amount of changes in expression levels of feature genes. Thus, inversely, the gene level difference between normal-class patterns and cancer-class patterns is a promising guidance to identify feature gene. The *p*-value in *t*-test between normal-class and cancer-class patterns is a more reliable guidance as it considers not only the level difference but also the significance of the difference. In [3], a gene is regarded as feature if the corresponding *p*-value is higher than a pre-determined cutoff value. Cao *et al*. [4] defined the relevance of a gene as the sensitivity of the output to the inputs in terms of the partial derivative. Guyon *et al*. in [5] defined the relevance of a gene in terms of its contribution to the cost function in Support Vector Machine (SVM). The corresponding gene ranking method names *Recurrsive Feature Elimination *(RFE). Several modifications on RFE, such as SQRT-RFE and Entropy-based RFE [6], were proposed to speed up the rank list construction process. Since the importance of variables is not assessed in the context of which other variables are not yet included, weaker subsets found by forward selection. Backward elimination method may outsmart it by eliminating the least promising variables and meanwhile providing the best classification from dependent variables (the variables that together perform best classification).

*Wrapper *is another approach to feature gene selection. In this approach, a feature gene set is found by optimizing certain measure quantities. Examples of these quantities include cross-validation [7] and bootstrap [8]. Shevade and Keerthi in [9] extracted feature gene by optimizing a SVM-liked energy function. Zhu *et al*. [10] presented a Markov blanket-embedded genetic algorithm (MBEGA) for gene selection problem. They used memetic operators to add or delete features (or genes) from a Genetic Algorithm (GA) solution in order to speed-up the GA convergence. Hong and Cho [11] enhanced the population divergence of a GA-based wrapper model by explicit fitness sharing. They also modified the representation of chromosome in GA to suit for large scale feature selection. Li *et al*. [12] presented a statistical approach for feature gene selection. Many subsets of genes that can well classify the training samples are identified; using GA, and the most frequently appeared genes in the subsets are then presumed as feature genes. Raymer *et al*. [13] reported a feature extraction algorithm to which feature selection, feature extraction, and classifier training are performed simultaneously, using a GA with the objective function involving training accuracy and the number of feature genes. Huerta *et al*. [14] suggested combining GA with SVM for the classification of microarray data. GA was used to evolve gene subsets, whereas SVM was used to evaluate the fitness values of the gene subsets in terms of classification accuracy. Shen *et. al*. in [15] reported a similar feature gene selection algorithm. It combined a discrete Particle Swarm Optimization (PSO) for search and SVM for fitness evaluation.

Gilad-Bachrach *et al*. [16] introduced a margin based feature selection criterion and applied it to measure the quality of a gene subset. A gene subset is said as optimal if the corresponding classifier has maximum error margin.

Most of the proposed feature selection algorithms [9-15] presume that the performance of feature gene set is associated with the training accuracy of the classifier built from it. However, since the number of training patterns related to the pattern dimension is small, training accuracy is not a representative performance measure. Alternatively, validation accuracy is a more objective and reliable performance measure. Though validation accuracy is never known in the training process, one can divide a training set of *n *samples into *m *non-overlapping subsets of roughly equal size; *m *- 1 of these subsets are combined as new training set and the remaining 1 subset is as validation set. The corresponding error is so-called cross-validated (CV) error. As noted by Ambroise and McLachlan [17], CV error may introduce a bias to the feature gene selection process. In addition, they proposed to tackle it (i.e. obtain an almost unbiased estimate) by a two-layered cross-validation approach. On the other hand, the validation accuracy relates to the generalization of a classifier whilst the generalization of a classifier is commonly measured from its error margin. It is reasonable to hypothesize that validation accuracy is proportional to the width of error margin. And it is worth to represent the performance of a feature gene set by its error margin.

In this paper, we proposed a novel feature gene extraction scheme, namely *Error-Margin Analysis *(EMA). EMA, as the name suggests, equates the performance of a feature gene set to error margin instead of classification accuracy. EMA starts from building an error margin curve representing error margin versus the number of mostly relevant genes. Afterwards, an analysis on the curve is performed to identify the optimal feature gene set. The proposed approach differs from [5] in the senses that the selection criterion is margin-based and parameter-less. It is also in contrast to [16], in which the feature genes are preferred to solely maximizing the error margin. Though [18] considers error margin in measuring the performance of a feature gene set, proper selections of penalty coefficient and the size value are critical. In summary, EMA has an advantage over [7-15] in measuring the performance of a feature gene set. Additionally, it is superior to [3-5] in the sense that the number of the feature genes extracted EMA is parameter-independent, whereas others are according to parameter settings.

EMA is based on two assumptions. It is assumes that 1) genes are independently expressed; 2) the distributions of gene expression are in Gaussian.

The rest of this paper is organized as follows: We first present an analysis on the relation between error margin and the number of feature genes. Afterwards, we proposed a novel feature gene extraction algorithm based on the error margin analysis. The experimental results are then reported and conclusions are drawn.

## Results

### Datasets

In this section, the performance of EMA is evaluated on three datasets:

#### i. Synthetic dataset

The **Synthetic **dataset acts as a control to check whether an algorithm underestimates or over-estimates the number of feature genes. It is assumed that the feature genes are distributed in Gaussian and the non-feature genes are uniformly and randomly distributed. Given an artificial pattern **x **= [*x*_1_, *x*_2_,..., *x*_500_] with the class *y*, the distribution *p*_*i*_(*x*) of the gene *x*_*i *_is shown in Table 1, where . It is suggested that an ideal feature selection algorithm should extract as many desired feature genes from the dataset as possible, in order to maximize the amount of possible pathways to the cancer diagnosis. Thus, the result on the **Synthetic **dataset indicates the ability of which the feature genes extracted by an algorithm cover the actual feature gene set. In this data set, each artificial pattern consists of 500 genes; the first 20 genes are assigned as desired feature genes and the remaining 480 genes are assigned as non-feature genes.

**Table 1:** The distribution of gene expressions in the synthetic dataset.

	y = -1	y = 1
i ∈ [1, 20]	*p*_*i*_(*x*) = *G*(*x *| *μ*_*i*_, *σ*_*i*_) where *μ*_*i *_= 0.3 - *i*0.05/20, *σ*_*i *_= 0.15 - *i*0.05/20	*p*_*i*_(*x*) = *G*(*x *| *α*_*i*_, *β*_*i*_) where *α*_*i *_= 0.7 - *i*0.05/20, *β*_*i *_= 0.15 - *i*0.05/20

i ∈ [21, 500]		

#### ii. Gastric cancer dataset [19]

This dataset shows expression levels of 123 samples (Osaka University Medical School Hospital). A hundred and twelve of them are normal-class patterns and the remaining twelve patterns are cancerous-class. It is available at the link: http://lifesciencedb.jp/cged/

#### iii. Oral cancer multiple datasets

We have available four microarray datasets; the first was measured with HG-U133 Plus2 and it has 11 normal and 50 cancerous samples, the second is from a HG-U133A and it has 22 normal and 22 cancerous samples, the third set comes from a HG-Focus and has only 22 cancerous samples and the fourth has 12 normal and 26 cancerous samples and measured also with HG-U133 Plus2. All the chips are manufactured by Affymetrix (Santa Clara, CA).

#### Algorithms for Comparison

To evaluate the impact of EMA, we compare its performance with five algorithms. The designs and settings of EMA and the algorithms for comparison are summarized below.

#### Test algorithm 1 - SVM with Feature Gene Extraction by Error Margin Analysis (SVM-ema)

SVM-ema estimates the number of feature genes *f*_0 _through the analysis on error margin. Given the gene relevance list, SVM-ema constructs the corresponding *error margin curve *and *f*_0 _is estimated as the critical point of the curve.

#### Test algorithm 2 - SVM with t-test based feature gene extraction (SVM-ttt)

In SVM-ttt [3], the relevance of a gene is measured on its *p*-value in *t*-test. A gene is indicated as a feature if its relevance is higher than a given cutoff *p*-value.

#### Test algorithm 3- SVM with Recursive Feature Elimination (SVM-rfe)

The gene relevance list is computed according to *recursive feature elimination *(RFE) [5]. At each iteration, RFE figures out and removes the least contributed gene from a set of considered genes. The iteration is repeated until all genes are removed from the set. The relevance of a gene is represented as the iteration index which it is removed. The curve representing the cross-validation error versus the number of mostly relevant features *f *is fitted by an exponential function *g*(*f*). The optimal number of feature genes is obtained as the value to which the change of *g*(*f*) is just smaller than threshold.

#### Test algorithm 4 - SVM with Margin-based Selection Criterion (SVM-msc)

SVM-msc [16] performs selection by search the feature gene set that maximizing a margin-based criterion.

#### Test algorithm 5 - Bayesian Logistic Regression (BLogReg)

BLogReg [20] is a gene selection algorithm based on sparse logistic regression (SLogReg). The regularization parameter arising in SLogReg is eliminated, via Bayesian marginalization, without a significant effect on predictive performance. The source code of BLogReg is taken from [21].

#### Test algorithm 6 - STW feature selection using generalized logistic loss (STW)

STW [22] was implemented exactly the same as SVM-RFE except that the hinge loss in SVM-RFE is replaced with the generalized logistic loss.

For SVM-ema, the parametric model *G*(.) for the estimation of LOOErM curve is chosen as second-order polynomial. The cutoff *p*-value of SVM-ttt is assigned as 0.005. For SVM-rfe, as suggested in [6], the threshold for obtaining the optimal number of feature is 0.0001 and the error is based on 3-fold experimental structure. The results of BLogReg and STW are obtained under the default parameters assigned in the corresponding source codes.

### Experiment Settings

For the **Synthetic **dataset, five hundreds patterns are generated in each run. Twenty five of them form training pattern set and the remaining four hundreds and seventy-five patterns form validation pattern set for performance measure. In each of the pattern sets, half of the patterns belong to negative class and another half belong to positive class.

For the **Gastric cancer **dataset, suppose *n*_- _is the number of normal-class patterns and *n*_+ _is number of cancer-class patterns in *T*, and *r *is the sampling rate, we randomly pick *rn*_+ _positive-class patterns and *rn*_- _negative-class patterns in *T *to form the training set. The remaining (1-*r*)*n*_+ _positive-class patterns and the remaining (1-*r*)*n*_- _negative-class patterns in *T *forms the validation set. The simulation is repeated with the sampling rate rising from 0.3 to 0.6.

For the **Oral Cancer **multiple datasets, the first three datasets form a superset *O*. Suppose *n*_- _is the number of normal-class patterns and *n*_+ _is number of cancer-class patterns in *O*, and *r *is the sampling rate, we randomly pick *rn*_+ _positive-class patterns and *rn*_- _negative-class patterns in *O *to form the training set. Meanwhile, the fourth dataset is regarded as the validation set. The corresponding accuracy represents the generalization ability of a test algorithm on the oral cancer classification problem. The simulation is repeated with the sampling rate rising from 0.1 to 0.7.

To provide a fair and repeatable comparison amongst the test algorithms, the performance of each test algorithm on a particular simulation is evaluated based on statistics obtained from 100 independent runs. For the Synthetic dataset, the patterns in both training set and validation set are randomly generated for each run. For the **Gastric cancer **dataset, the substituted random number is regenerated for each particular invalid expression in each pattern. For **Oral cancer **multiple datasets, the patterns in the training set are randomly re-picked for each run. All test algorithms are implemented in MATLAB language.

## Simulation Results

### Synthetic dataset

Table 2 lists the statistics of the numbers of the feature genes extracted by the test algorithms. Table 3 lists the statistics of the validation accuracies of the test algorithms. The values inside blankets represent the averaged number of actual feature genes (i.e. the 20 predefined feature genes) extracted by the corresponding algorithms. The averaged numbers of feature genes extracted by SVM-ema, SVM-ttt, SVM-rfe, SVM-msc, BLogReg and STW are 17.9, 43.27, 65.38, 448, 2.45 and 45.42 respectively. Though BLogReg extracted the smallest amount of feature genes, it ranks the last on the accuracy measure. The averaged accuracies of BLogReg and STW are 50% and 93.79% respectively; the remaining algorithms are with 100% averaged accuracies. On average 16.63 out 17.9 genes extracted by SVM-ema are actual feature genes. The averaged number of actual feature genes extracted by SVM-ttt, SVM-rfe, SVM-msc, BLogReg and STW are 19, 19, 18.93, 1 and 8.51 respectively.

**Table 2:** The statistics of the numbers of feature genes extracted by the test algorithms: Synthetic dataset.

	Mean	Std.	Median	Min.	Max.
**SVM-ema**	17.90 (16.63)	1.40	18.00	15.00	23.00
**SVM-ttt**	43.27 (19.00)	4.72	43.00	31.00	56.00
**SVM-rfe**	65.38 (19.00)	4.62	66.00	50.00	75.00
**SVM-mcs**	448.01 (18.93)	36.92	455.50	356.00	500.00
**BLogReg**	2.45 (1.00)	0.56	2.00	2.00	4.00
**STW**	45.42 (8.51)	6.24	47.00	16.00	48.00

The values inside blankets represent the averaged number of actual feature genes extracted by the corresponding algorithms.

**Table 3:** The statistics of the validation accuracies of the test algorithms: Synthetic dataset.

	Mean	Std.	Median	Min.	Max.
**SVM-ema**	100.00%	0.00%	100.00%	100.00%	100.00%
**SVM-ttt**	100.00%	0.00%	100.00%	100.00%	100.00%
**SVM-rfe**	100.00%	0.00%	100.00%	100.00%	100.00%
**SVM-mcs**	100.00%	0.00%	100.00%	100.00%	100.00%
**BLogReg**	50.00%	0.02%	50.00%	50.00%	50.22%
**STW**	93.79%	7.71%	97.56%	64.44%	100.00%

### Gastric cancer dataset

**Figure 1:**
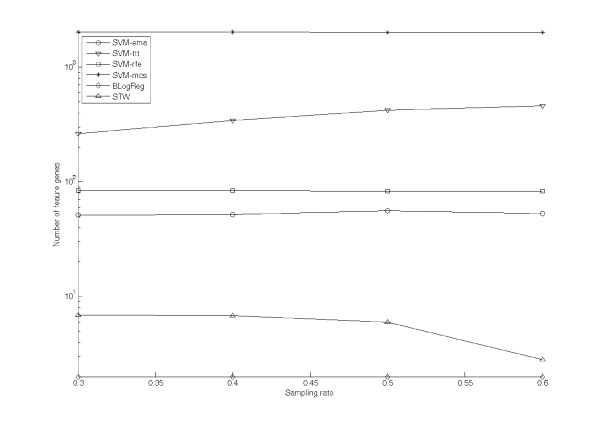
**The number of feature genes extracted from Gastric cancer dataset by test algorithms**.

Figure 1 shows the averaged numbers of feature genes extracted by the test algorithms against the sampling rate *r *ranging from 0.3 to 0.6. The *y*-axis of the figure is in log scale. The results of SVM-ema, SVM-ttt, SVM-rfe, SVM-mcs, BLogReg and STW are represented by the lines with the markers 'O', '∇', '▽', '*', '◊' and 'Δ' respectively. Seen from the figure, as the sampling rate increases, the number of feature genes *f*_*ttt *_extracted by SVM-ttt increases from 263.1 at *r *= 0.3 to 458.9 at *r *= 0.6, which is approximately linearly proportional to *r*. For SVM-rfe, the number of extracted feature genes *f*_*rfe *_slightly increases from 79.5 at *r *= 0.3 to 84.7 at *r *= 0.6. For SVM-ema, the number of feature genes *f*_*ema *_is insensitive to *r*. It is in the range [51.4, 55.8]. For SVM-mcs, the number of extracted feature genes *f*_*mcs *_is again insensitive to *r *but constantly stay at a large value ranging in [2002.7, 2033]. In contrary to SVM-mcs, BLogReg constantly selects small set of feature genes; the corresponding number of extracted feature genes *f*_*BLR *_is in a small range from 2 to 3. Interestingly, the number of extracted feature gene *f*_*STW *_by STW is inversely proportional to the sampling rate. The value of *f*_*STW *_decreases from 6.85 at *r *= 0.3 to 2.83 at *r *= 0.6.

**Figure 2:**
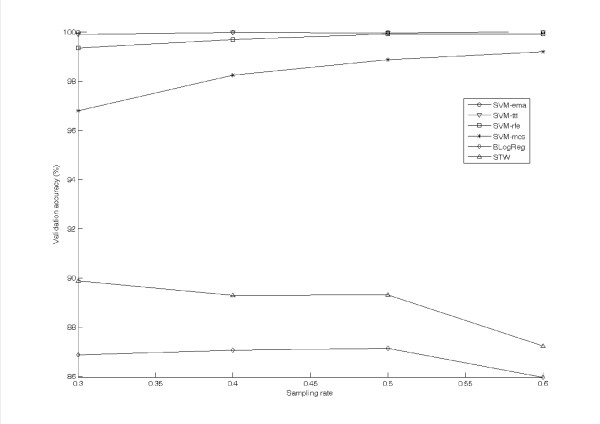
**The validation accuracies evaluated on Gastric cancer dataset by test algorithms**.

Figure 2 shows the averaged validation accuracy of the test algorithms against the sampling rate *r *varying from 0.3 to 0.6. The results of the test algorithms are represented by the lines with the same markers in Figure 1. Seen from the figure, SVM-ema, SVM-ttt and SVM-rfe constantly and accurately classify the validation set, the corresponding accuracies range from 99.34% to 100.0%. The validation accuracy of SVM-mcs is just slightly lower than those of the above three algorithms. It ranges from 96.8% at *r *= 0.3 to 99.19% at *r *= 0.6. On the other hand, the validation accuracies of BLogReg and STW decrease along with *r*. The accuracies of BLogReg and STW are in the ranges [85.96%, 87.14%] and [87.23%, 89.88%] respectively.

### Oral cancer multiple datasets

**Figure 3:**
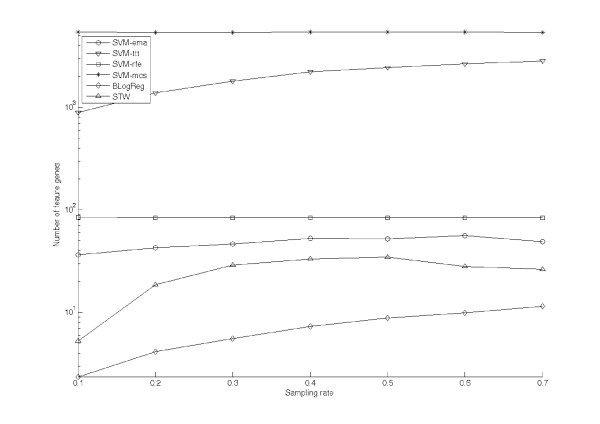
**The number of feature genes extracted from Oral cancer dataset by test algorithms**.

Figure 3 shows the averaged numbers of feature genes extracted by the test algorithms against the sampling rate *r *ranging from 0.1 to 0.7. The *y*-axis of the figure is again in log scale. Similar to Figure 1 and Figure 2, the results of SVM-ema, SVM-ttt, SVM-rfe, SVM-mcs, BLogReg and STW are represented by the lines with the markers 'O', '∇', '▽', '*', '◊' and 'Δ' respectively. Seen from the figure, for SVM-ema, SVM-ttt, SVM-rfe and SVM-mcs, the influences of the sampling rate to the number of feature genes are similar to those on the **Gastric cancer **dataset: As the sampling rate increases, the value of *f*_*ttt *_linearly increases from 890.9 at *r *= 0.1 to 2826.3 at *r *= 0.7; the value of *f*_*rfe *_is insensitive to *r *and is in the range [81.5, 88.4]; the value of *f*_*ema *_slightly increases from 36.5 at *r *= 0.1 to 56.48 at *r *= 0.7; the value of *f*_*mcs *_is again insensitive to *r *but constantly stay at large values ranging in [5367, 5458]. Comparing between SVM-ema and SVM-rfe, though the grow rate of *f*_*ema *_is large than that of *f*_*rfe*_, *f*_*ema *_is consistently lower than *f*_*rfe*_. And it is also significantly lower than *f*_*ttt *_and *f*_*mcs*_. For BLogReg, the number of extracted feature genes is yet in low range from 2.37 to 11.53. The value of *f*_*STW *_increases from 5.31 at *r *= 0.1 to 34.6 at *r *= 0.7.

**Figure 4:**
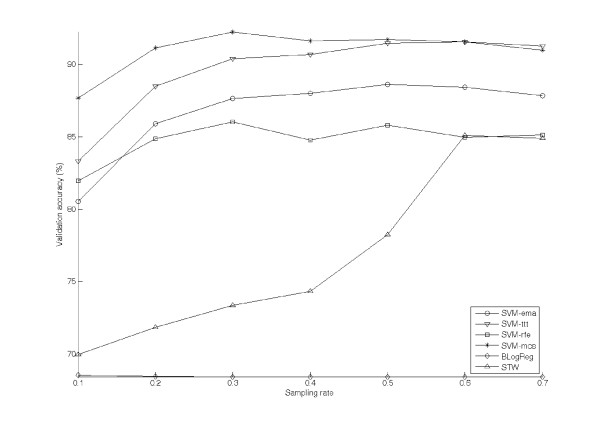
**The validation accuracies evaluated on Oral cancer dataset by test algorithms**.

Figure 4 shows the averaged validation accuracy of the test algorithms against the sampling rate r varying from 0.1 to 0.7. The results are represented by the lines with the same markers in Figure 3. Seen from the figure, with the exception of BLogReg and STW, the validation accuracies of the test algorithms slightly increase along with r. The range of the accuracy of SVM-ttt is [83.32%, 91.58%]. For SVM-ema, its accuracy ranges from 80.5% to 88.6%. The validation accuracies of SVM-rfe and SVM-mcs are in the ranges [81.9%, 86.0%] and [87.7%, 92.26%] respectively. For BLogReg, its validation accuracy is insensitive to the sampling rate; the accuracy keeps at a low value ranging from 68.42% to 68.53%. In contrary to BLogReg, the validation of accuracy of STW is much affected by the size of training set. When the value of r is in between of 0.3 and 0.4, the corresponding accuracy is at a relatively low value ranging from [69.95%, 85.11%]. As the value of r reaches 0.6, the accuracy of STW increases to the same of SVM-rfe but is yet lower than that of SVM-ema.

## Discussion

Providing a cancer disease correlates to certain amount of genes (namely *the actual feature genes*), an ideal feature selection algorithm can extract this set of genes from training set without over-extracting the irrelevant genes or filtering-out some of *the actual feature genes*. The ideality is due to the fact that as more *actual feature genes *are extracted, the more pathways are provided to the cancer diagnosis. Thus, under a controlled environment, it is suggested to measure the performance of an algorithm according to the ratios between the number of feature genes *f *extracted by this algorithm, the number of actual feature genes *f*_*A *_extracted, and the number of actual feature genes *f*_*0*_. The so called hitting rate *r*_*h *_of this algorithm is defined as *f*_*A*_/*f*_*0*_; and the missing rate *r*_*m *_is defined as (*f *- *f*_*A*_)/*f*. The algorithm *J *is suggested to be superior to another algorithm *K *if *r*_*h*_(*J*) is larger than *r*_*h*_(*K*) and *r*_*m*_(*J*) is smaller than *r*_*m*_(*K*). Table 4 lists the hitting rates and the missing rates of the test algorithms measured on the synthetic dataset. Seen from the table, SVM-ttt, SVM-rfe and SVM-mcs are with high hitting rates but also high missing rates, which infer there are over-extractions of the features. Alternatively, it is suggested that STW underestimates the number of features as its relatively low hitting rate. Moreover, BLogReg extremely underestimates the number of features as its unusual low hitting rate, i.e. 5%. In general, SVM-ema is superior to other algorithms as its hitting rate is high and missing rate is low. The results show that SVM-ema can extract the most relevant set of feature genes.

**Table 4:** The average hitting rate *r*_*h *_and the average redundancy rate *r*_*r *_of the test algorithms: Synthetic dataset.

	SVM-ema	SVM-ttt	SVM-rfe	SVM-mcs	BLogReg	STW
*r*_*h*_	83.15%	**95.00%**	**95.00%**	94.65%	5.00%	42.55%
*r*_*r*_	**7.09%**	56.09%	70.94%	95.77%	59.19%	81.27%

For the cases of two real datasets, Figure 1 and Figure 3 indicate the number of feature genes extracted by different algorithms. We found that SVM-ema, SVM-rfe and SVM-mcs are insensitive to the sampling rate, for which the numbers of feature genes just slightly increase along with the sampling rate *r*. Though SVM-ema and SVM-mcs both employ error margin on their gene selection criterions, SVM-ema consistently result in much less number of feature genes. As indicated in previous sections, irrelevant genes may also contribute to the error margin. The maximization approach of SVM-mcs tends to extract as more genes as possible. Thus, SVM-mcs overextracts feature genes in order to achieve larger error margin. Seen from Figure 1 and Figure 3, the numbers of feature genes extracted by SVM-mcs are unusually large: For the **Gastric cancer **dataset, the minimal number is 2002, for which nearly 99% genes are regarded as feature. For the **Oral cancer **data, the number is more than 5000, in which nearly 87% genes are considered as features. Comparing to the results of SVM-ema, the number of feature genes extracted by SVM-mcs is around 35 times and 149 times more than that of SVM-ema for the **Gastric cancer **dataset and the **Oral cancer **datasets respectively. The reason of this difference is that EMA is able to decompose the contributions of the feature genes from those of the background genes. This also indicates that purely maximizing error margin is not a practical selection criterion.

While comparing the validation accuracies amongst the test algorithms, SVM-ttt and SVM-mcs should be ignored as their high accuracies are archive by overextracting feature genes. Seen from the results shown in Figure 2 and Figure 4, the performance of SVM-ema is better than that of SVM-rfe in terms of not only the validation accuracy but also the number of feature genes. SVM-ema is also superior to BLogReg and STW. This superiority of SVM-ema suggests that 1) margin-based criterion is more suitable to represent the performance of a feature gene set; and 2) this criterion is more robust than those of BLogReg and STW in the sense that BLogReg and STW may under-estimate the number of feature genes.

## Conclusions

This paper proposes a feature extraction algorithm of error margin analysis that uses margin-based criterion to measuring the quality of a feature set. Error margin is a better indicator than training accuracy in representing the generalization ability of a classifier. However, maximizing the error margin may lead to overextraction of features. Therefore, we propose to make a tradeoff between the performance and the number of features, which is done by analyzing the curve of error margin. Under the assumptions on gene independency and on gene distribution, the analysis shows that the error margin of only involving the relevant genes grow faster than that of involving random genes. Based on this observation, we model the extraction process as an estimation of critical point in the *error margin curve *of error margin versus the number of mostly relevant genes. Compared with existing algorithms that use either margin-based selection criterion or "filtering" approach, our algorithm has distinct advantage, which has been proven from theoretical framework.

Computational experiments of comparing EMA with other approaches including wrapper models and filtering models. The experimental results show that:

1) Error margin is a more representative measure to the generalization ability of a classifier than training accuracy;

2) Solely maximizing error margin may lead to overextraction of features;

3) SVM-ema can make right balance between the performance and the size of resultant feature gene set.

Possible future works include 1) an analysis on the *error margin curve *when the gene distribution is non-Gaussian, 2) deriving a more accurate parametric model for the margin curve segments *w*_*I *_and *w*_*R *_and 3) an extension to the analysis on error margin of non-linear classifier.

## Methods

### Error-Margin as an Indicator to Feature Genes

Gene expression level difference and the *p*-value of the expression level are promising relevance measures of a gene. The gene rank list sorted according to these measures provides guidance for feature gene selection. On the other hand, margins play a crucial role in modern machine learning research. It represents the generalization ability of a classifier or the confidence of the decision made by the classifier. It is valuable to investigate the possibility of which uses error margin as a criterion to decide how many genes should be selected from the list. In this section, an analysis on the relation between error margin and the number of mostly relevant genes is presented.

Given a training set *S *= {[**x**_**j **_| *y*_*j*_]} where **x**_**j **_∈ *X *⊆ ℜ^*d *^and *y*_*j *_∈ {-1, 1}, and [**u **∈ ℜ^*d*^, *λ*] is the decision hyperplane of *S *obtained by SVM, the corresponding error margin *w *is defined as:(1)

where {*h*_*i*_} are constants, {*x*_*j*, *i*_} and hence {*v*_*j*_} are random variables.

Suppose *C*_- _contains the indices of all normal-class patterns (i.e. *y*_*j *_= -1 for *j *∈ *C*_-_) in *S *and *C*_+ _contains the indices of all cancer-class patterns (i.e. *y*_*j *_= 1 for *j *∈ *C*_+_) in *S*, since SVM guarantees that the error margin is maximal, the minimal error margin amongst the normal-class patterns equals to that amongst the cancer-class patterns:(2)

In the rest of this paper, the analysis considers the minimal error margin amongst of the cancer-class patterns {*v*_*j*_} for *j *∈ *C*_+_.

**Figure 5:**
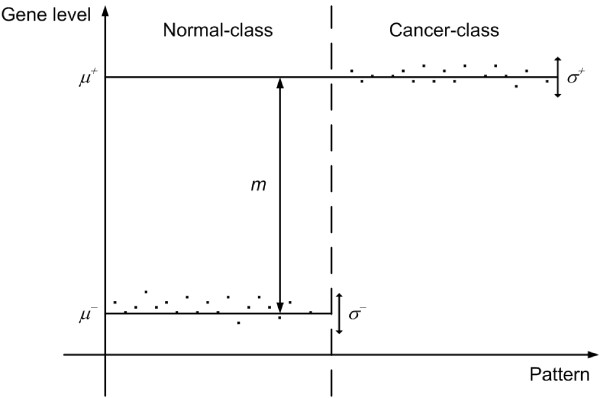
**General classification model based on gene expression level**.

We start the error margin analysis by studying the distribution of error margin of a training pattern. The first assumption made in this analysis is that the probability density function *q*_*i*_(*x*) of the *i*^th ^gene *x*_*i *_is Gaussian:(3)

where  and . Figure 5 shows the general classification model of gene expression level.  and  shown in the figure represent the mean and the variance of the *i*^th ^gene amongst the patterns in *C*_-_. (the physical meanings of  and the level difference *w*_*i *_of the *i*^th ^gene). One important assumption of gene pattern in bioinformatics is that the level difference *m*_*R *_for relevant (feature) gene is much larger than the level difference *m*_*I *_for irrelevant (non-feature) gene, i.e. *m*_*R *_>>*m*_*I*_.

**Figure 6:**
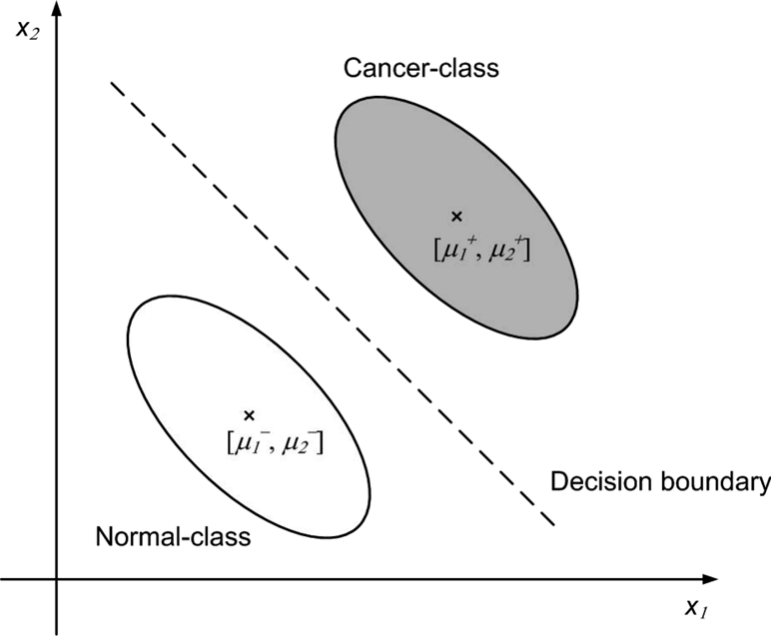
**A two-dimensional example of pattern translation: before translation**.

**Figure 7:**
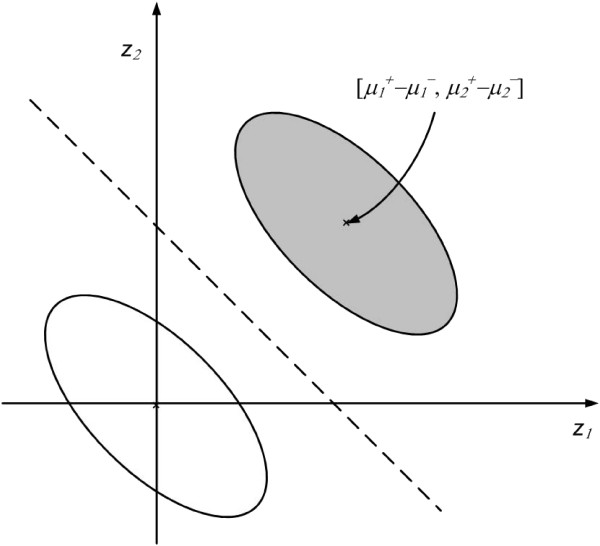
**A two-dimensional example of pattern translation: after translation**.

Since *w *is translation-invariant, we translate the gene pattern **x**_**j **_∈ *X *to **z**_**j **_= **x**_**j **_- ***μ***_- _∈ *Z *for all *j *where . Meanwhile, the decision hyperplane of *S *in *Z *is transformed as [**h**, *b*] = [**u **- ***μ***_**-**_, *λ*+**u**·**μ**_**-**_]. Figure 6 and Figure 7 show a 2-dimensional example of the pattern translation.

Figure 6 shows the original 2-dimensional feature space *X*. The white ellipse represents the region of normal-class patterns whilst the grey-filled ellipse represents the region of the cancer-class patterns. The center of the normal-class patterns is , whereas the center of the cancer-class patterns is . The dotted line represents the decision hyperplane obtained by SVM. Figure 7 shows the translated feature space *Z*. The centers of the normal-class patterns and of the cancer-class pattern are translated to [0, 0] and  respectively.

After the translation, the probability density function *r*_*i*_(*z*) of *z*_*i *_is:(4)

Since  is the level difference *m*_*i *_of the *i*^th ^gene, the eq. (4) can be further expressed as:(5)

Let *a*_*i *_= *h*_*i*_*z*_*i*_, the corresponding probability density function *p*_*i*_(*a*) is expressed as:(6)

At this stage, we made the second assumption that genes {*z*_*i*_}, and hence {*a*_*i*_}, are independent. Under this assumption, the probability density function *p*_*v*_(*v*) of *v *= ⟨**h, z**⟩ + *b *appeared in eq. (2) can now be expressed as:(7)

where 

Since the convolution of two Gaussian functions is still a Gaussian function:(8)

where

*p*_*v*_(*v*) can be simplified as:(9)

where  and .

The analysis on the relation between error margin and the number of mostly relevant genes can be divided into three cases:

### Case 1: Linearly separable training set with zero gene variance

It is commonly to assume that microarray pattern set is linearly separable. The linear separability of a pattern set is discussed at Appendix I. When the training set is linearly separable, the probability of which *w *is lower than a given value *w*_*0 *_is described by the function:(10)

where *n*_+ _is the cardinality of *C*_+_. The probability density function *p*_*w*_(*w*) of *w *is:(11)

The expected error margin  for linearly separable training set is:(12)

where *η*(.) is monotonic increasing and depends on on *n*_+_. The details of eq. (12) can be found in Appendix II.

A pattern set is said as ideal if the gene variance approach to zeros, i.e. *σ*_*i *_→ 0, for all *i *∈ [1, *d*]. For such case, *p*_*w*_(*w*) can be simplified as *p*_*v*_(*w*).(13)

and the expected error margin  is computed as a weighted sum of the expected gene level differences {*m*_*i*_}_*i*∈[1, *d*]_:(14)

Given a gene relevance list *L *= {*ϕ*_*i*_} where a gene is at a former position of the list if it has higher relevance, we define , as the expected error margin when the *i *mostly relevant genes are considered:(15)

In this paper, the term *error margin curve W*(*i*) refers to the curve representing error margin versus the number of mostly relevant genes, i.e. *W*(*i*) = .

**Figure 8:**
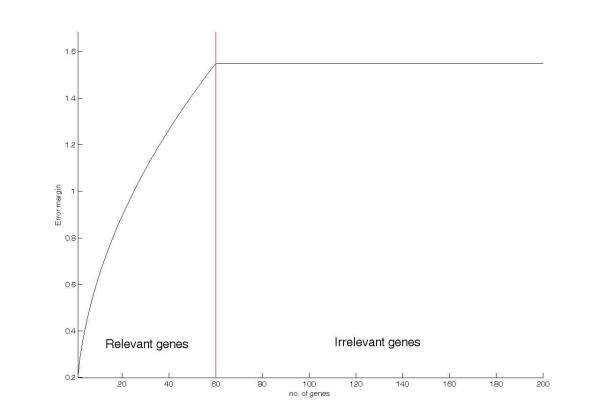
**A typical *error margin curve *for ideal pattern set**.

Suppose there are *n*_*R *_feature genes (i.e. the *n*_*R *_mostly relevant genes are the feature genes), the *error margin curve *can be divided into two segments: 1) the relevant gene segment *W*_*R*_(*i*) for *i *∈ [1, *n*_*R*_] and 2) the irrelevant gene segment *W*_*I*_(*i*) for *i *∈ [*n*_*R *_+ 1, *d*]. As we hypothesize that *m*_*R *_is significantly larger than *m*_*I*_, in addition to that the expected error margin is a weighted sum of the gene level differences of the considered genes, the averaged grow rate of *W*_*R*_(*i*) must be higher than that of *W*_*I*_(*i*). Thus, there should be a critical point on the *error margin curve*, and this point indicates the boundary between relevant genes and irrelevant genes. Figure 8 shows a typical *error margin curve *for ideal pattern set. Seen from the figure, the critical point of the curve is at the boundary between the relevant and the non-relevant genes. In other words, the estimation of the number of feature genes is equivalent to find the critical point on the *error margin curve*.

### Case 2: Linearly separable training set with non-zero gene variance

**Figure 9:**
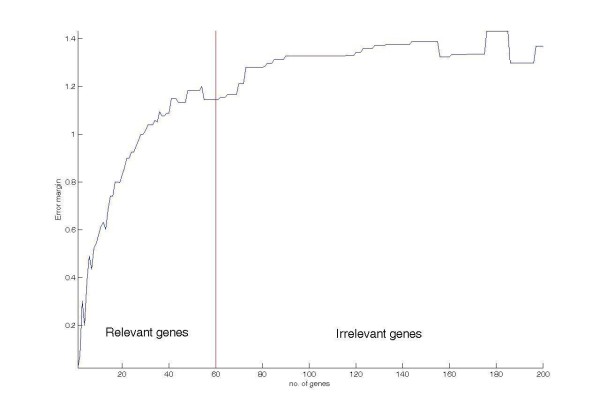
**A typical *error margin curve *for case of non-zero variance**.

For the case of which the training set is linearly separable but *σ*_*i *_> 0, the influence of *σ*_*i *_to the *error margin curve *can be expressed as follows: When *σ*_*i *_increase, gene patterns spread wider in *X *and they have higher chance to get closer to the decision hyperplane. Thus, a narrower error margin is expected. Furthermore, when more genes are considered, *m*_*w *_and *σ*_*w *_in *p*_*v*_(*v*) grow in different rates, in which , as a weight sum of *m*_*w *_and *σ*_*w *_according to the eq. (12), is neither monotonic increasing nor monotonic decreasing. Therefore, the *error margin curve *for *σ*_*i *_> 0 is filled with small oscillation. Figure 9 shows a typical *error margin curve *for *σ*_*i *_> 0.

### Case 3: Linearly non-separable training set

In case of linearly non-separable training set, the soft-margin idea choose a decision hyperplane that the classification accuracy is as high as possible, while still maximizing the error margin of the correctly-classified pattern set *V' *∈ . Thus, the *error margin *in this case is measured from *V'*. Since the excluded patterns from *V' *are those with minimal (and negative) error margin *v*_*i*_, it is expected that 1) the mean of *V' *is larger than that of *V *and 2) the variance of *V' *is smaller than that of *V*. Under a practical assumption that the gene distributions in *V' *are also Gaussian, the soft-margin idea brings the error margin analysis of linearly non-separable training set back to the case of linearly separable pattern set.

In summary, when a training set is linearly separable and *σ*_*i *_= 0 for all *i*, the critical point of the *error margin curve *is definitely the boundary point between relevant and irrelevant gene sets. However, if 1) *σ*_*i *_> 0 for at least one gene and/or 2) the training set is linearly non-separable, oscillation is introduced to the curve and blunts the critical point. For such case, feature gene extraction is modeled as the estimation of critical point of the error margin.

### Feature Gene Extraction by Error-Margin Analysis

In this section, we report a novel feature gene extraction algorithm, namely *Feature Gene Extraction by Error Margin Analysis *(EMA). Based on the error margin analysis presented in the previous section, the feature gene extraction can be modeled as the search for the critical point of the *error margin curve*.

In order to moderate the dependency of error margin on pattern set, *Leave-One-Out Error margin *(LOOErM) is used. LOOErM, as the name suggests, leaves a single pattern from the training set and compute the error margin of the decision hyperplane defined by the remaining patterns. This is repeated such that each pattern in the training set is left once. For a training set *S *consisting of *n *patterns, *n *error margins {*g*_*j*_}_*j*∈[1, *n*] _are obtained. The LOOErM of *S *is defined as the average of {*g*_*j*_}. *Algorithm A1 *summarizes the procedure of LOOErM.

#### Algorithm A1: Leave-One-Out Error Margin

Input: 1) Pattern set *S *= {[**x**_**j **_| *y*_*j*_}]}_*j *∈ [1, *n*]_, 2) the index set of the considered genes *F*

1.   **For ***j *: = 1 **to ***n*

1.1      Define the pattern subset *Z *= {[**x**_**k**_(*i*)_*i*∈*F *_| *y*_*k*_]}_*k*≠*j*_

1.2      Train SVM on *Z*: the corresponding decision hyperplane denotes by *H*_*j*_(**z**): ⟨**h**·**z**⟩ + *b *where ⟨**a**·**b**⟩ is the dot-product of the vectors **a **and **b**.

1.3      Compute the error margin *g*_*j *_of *H*_*j*_: 

2.      **Next ***j*

3.      

Output: the leave-out-one error margin 

Since the *error margin curve *is filled with small oscillation due to gene variations amongst patterns, the critical point of the curve is not as significant as that shown in Figure 7. Thus, a noise reduction on the *error margin curve *is necessary. It can be done by fitting a parametric function *G*(*i*|**α**) to the curve. Recalling that the *error margin curve *is composed of two segments: *W*_*R *_for relevant genes and *W*_*I *_for irrelevant genes, the estimation (*i*) of *W*(*i*) consists of two parts: *G*(*i*|**α**_**R**_) and *G*(*i*|**α**_**I**_). The first part deals with the noisy *W*_*R *_whilst the second part deals with the noisy *W*_*I*_, i.e. *W*_*R*_(*i*) ≈ *G*(*i*|**α**_**R**_) and *W*_*I*_(*i*) ≈ *G*(*i*|**α**_**I**_). In addition, since the *error margin curve *is expected as a continuous function, *W*_*R *_should meet *W*_*I *_at the critical point *c*, i.e. *G*(*c*|**α**_**R**_) = *G*(*c*|**α**_**I**_). In a whole say, the *error margin curve *can be estimated as:(16)

and the corresponding estimation error *ε *is defined as:(17)

Seen from eq. (17), *ε *naturally depends on *c*, **α**_**R **_and **α**_**I**_. In other words, the performance of an arbitrary critical point *c *= *f *can be represented by the error . Given that *G*(.) is sufficient to model *W*_*R *_and *W*_*I*_, the optimal critical point *f*_*0 *_of the *error margin curve *is defined as the critical point where the estimation error of (*i*) is minimum, i.e. .

Given a training set *S *= {[**x**_**j **_= [*x*_*j,1*_, *x*_*j,2*_,..., *x*_*j,d*_] ∈ ℜ^*d *^| *y*_*j *_∈ {-1, 1}]}_*j*∈[1, *n*]_, we first rank the genes according to their relevancies. We denote *L *= {*ϕ*_k_}_*k *= 1,2,..., *d *_as the gene relevance list to which the relevance of the *ϕ*_a_^th ^gene is larger or equals to that of the *ϕ*_b_^th ^gene for all *a *<*b*. The list *L *is then used to rearrange *S *as {[**x**_**j**_(*L*) | *y*_*j*_]}_*j*∈[1, *n*]_. Afterwards, we compute the *error margin curve W*(*i*) =  where  is the LOOErM computed from *Algorithm A1 *with *F *= {1, 2,..., *i*}.

In this paper, *G*(.) is chosen to be a polynomial function. The corresponding estimation error *ε*_*f *_for an arbitrary critical point *c *= *f *can be obtain by the least square method. The details of the method can be found in Appendix III. As benefitted from the prior-knowledge that the number of feature genes is commonly lower than a pre-determined value *f*_*max*_, say for example *f*_*max *_= 100, we only need to examined the estimation errors up to first *f*_*max *_mostly relevant genes, i.e. {*ε*_*f*_} for *f *∈ [1, *f*_*max*_]. The optimal critical point *f*_*0 *_is estimated as the one with minimum estimation error, i.e. , and the index set of the feature gene *F*_*0 *_is . *Algorithm A2 *summarizes the procedure of *Feature Gene Extraction by Error-Margin Analysis*.

#### Algorithm A2: Feature Gene Extraction by Error-Margin Analysis

*Input*: 1) Pattern set *S *= {[**x**_**j **_= [*x*_*j,1*_, *x*_*j,2*_,..., *x*_*j*, *d*_] ∈ ℜ^*d *^| *y*_*j *_∈ {-1, 1}]}_*j*∈[1, *n*]_, 2) maximum number of considered genes *f*_*max*_, 3) parametric error margin model *G*(.)

   /* Construct the gene relevance list *L*: BEGIN */

1.   Compute the relevance *r*_*i *_of the *i*^th ^gene:

where Ω(*A*, *B*) is the *p*-value of two point sets *A *and *B*, *C*_- _contains the indices of all normal-class patterns in *S *and *C*_+ _contains the indices of all cancer-class patterns in *S*.

2.   Define the gene relevance list *L *= {*ϕ*_*j*_}_*j *= 1,2,..., *d *_where the relevance of the *ϕ*_a_^th ^gene is larger or equals to that of the *ϕ*_b_^th ^gene, i.e.  for all *a *<*b*.

3.   Rearrange the gene order of *S *according to *L*: *S *← {[**x**_**j**_(*L*) | *y*_*j*_]}_*j*∈[1, *n*]_

   /* Construct the gene relevance list *L*: END */

   **/* Construct the LOOErM curve {}: BEGIN */**

4.   **For ***i *: = 1 **to ***f*_*max*_

4.1      Compute  by *Algorithm A1 *where the set *F *used in the algorithm is defined as {1, 2,..., *i*}.

5.   **Next ***i*

   /* **Construct the LOOErM curve **{}: END */

   /* Search for the critical point of the LOOErM curve: BEGIN */

6.   **For ***f *: = 1 to *f*_*max*_

6.1      Compute the estimation error . If *G*(.) is a polynomial function, the optimal **α**_**R **_and **α**_**I **_can be found by the method listed in Appendix III.

7.   **Next ***f*

8.   Compute the optimal critical point *f*_*0 *_as arg 

   /* Search for the critical point of the LOOErM curve: END */

*Output*: The index set of the feature genes 

**Figure 10:**
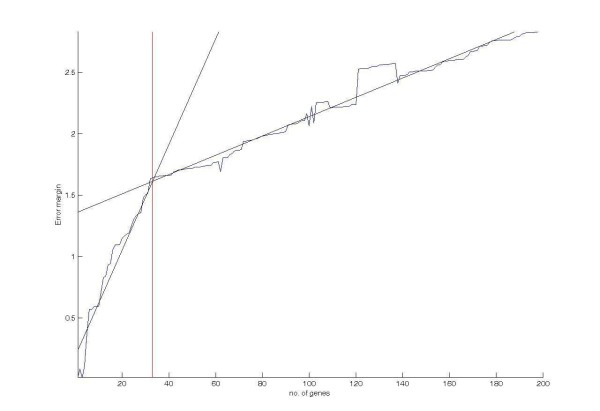
**Gene extraction by error margin analysis on the Gastric cancer dataset**.

**Figure 11:**
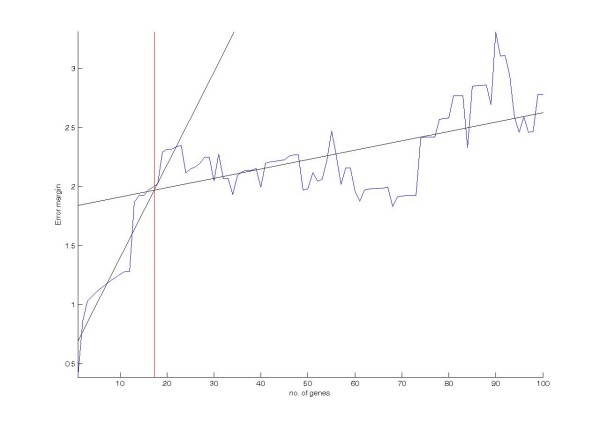
**Gene extraction by error margin analysis on the Oral cancer dataset**.

Figure 10 and Figure 11 show two examples of feature gene extraction by error margin analysis. For each example, the blue curve represents the *error margin curve*. The black lines represent the parametric estimations of the curve segments *W*_*R *_and *W*_*I*_. The red line represents the boundary between the relevant genes and the irrelevant genes, which passes through the intersection of the black lines. Figure 10 illustrates the gene extraction on the **Gastric cancer **dataset whilst Figure 11 illustrates the gene extraction on **Oral cancer **multiple datasets. The details of the datasets can be found in the experimental result section. Seen from the figure, each of the *error margin curve*s composes of two line segments and they grow in different rates.

## Authors' contributions

HLZ and CKC designed the methodology, devised the study and prepared the manuscript. CKC performed the data analysis and organized the experimental results. WPK designed the microarray experiment of the oral cancer. JL performed the microarray experiment of oral cancer in WPK's lab. All authors read and approved the final manuscript.

## Appendix I

### Linearity of Gene Patterns

Given a pattern set *S *= {[**x**_**j **_= [*x*_*j,1*_, *x*_*j,2*_,..., *x*_*j*, *d*_] ∈ *X *⊆ ℜ^*d *^| *y*_*j *_∈ {-1, 1}]}_*k*∈[1, *n*] _where the first *n*_+ _patterns is positive-class and the remaining *n*_- _= *n *- *n*_+ _patterns is negative-class patterns, if there exisit a *d *by *n *transformation matrix **T **such that every pattern **x**_**j **_is transformed to a point **x**_**j**_' in *n*-dimensional Euclidean space, **x**_**j**_**T **= **x**_**j**_' = :(18)

We must be able to found at least one decision hyperplane **h**_**I**_, for example  such that the transformed patterns {**x**_**j**_'} can be linearly separable:(19)

Since **T **is a linear transformation, the eq. (19) can be rewritten as(20)

Eq. (20) infers that *S *can be linearly separated by the hyperplane **Th**_**I**_. In conclusion, *S *is linearly separable if the transformation matrix **T **exists.

#### Existence of the transformation matrix

According to eq. (18), **T **is defined as the right inverse of **P**, which can be decomposed as **P**^**T**^(**PP**^**T**^)^-1^. Thus, **T **exists if **P **has the rank *m*.

When the number of genes *d *is much larger than the number of training patterns *n*, i.e. *d *>>*n*, the probability of that **T **exists is higher. Reminding that gene pattern analysis deals with small sample size and high sample dimension, the existence of T can be easily archived. Thus, gene patterns are reasonably assumed to be linearly separable. Additionally, since support vector machine guarantees that the decision hyperplane has maximum error margin, linear SVM model is ideal for gene pattern classification.

## Appendix II

Study of the expected error margin  for linearly separable training set:(21)

Considering the first integration part of eq. (21)(22)

Let  and *dw *= *σ*_*w*_*dz*. Additionally, *z *= -∞ when *w *= -∞ and *z *= ∞ when w = ∞. Thus, eq. (22) is transformed as:(23)

We further let , *t *= *yσ*_*w *_+ *m*_*w *_and *dt *= *σ*_*w*_*dy*. Additionally, *y *= -∞ when *t *= -∞ and *y *= *z *when *t *= *z*σ_*w *_+ *m*_*w*_. Thus, eq. (23) is further transformed as:

Therefore, the expected error margin for linearly separable training set is:(24)

## Appendix III

Suppose *G*(.) is a *γ*-order polynomial, the estimations of *w*_*R *_and *w*_*I *_are *G*(*x *| **α**_**R **_= [**A**, *B*]) =  and  respectively

Since the estimations are subjected to the condition:(25)

The estimation error *ε *of  can be rewritten as:

The optimal values of **A**, **B **and **C **can be computed from the least square method. Firstly, we set the derivative of *ε *with respect to {*A*_*k*_}_*k*∈[1, *γ*]_, C and {*C*_*k*_}_*k*∈[1, *γ*] _as zeros:

Afterwards, we define the matrices M and Y:

where

The optimal parameter vector Ψ = [**A *B *C**]^T ^is computed as Ψ = **M**^**-1**^**Y **and the optimal value of *D *can be found by the eq. (25).
